# High neutralizing antibody titer in intensive care unit patients with COVID-19

**DOI:** 10.1080/22221751.2020.1791738

**Published:** 2020-07-20

**Authors:** Li Liu, Kelvin Kai-Wang To, Kwok-Hung Chan, Yik-Chun Wong, Runhong Zhou, Ka-Yi Kwan, Carol Ho-Yan Fong, Lin-Lei Chen, Charlotte Yee-Ki Choi, Lu Lu, Owen Tak-Yin Tsang, Wai-Shing Leung, Wing-Kin To, Ivan Fan-Ngai Hung, Kwok-Yung Yuen, Zhiwei Chen

**Affiliations:** a AIDS Institute, Li Ka Shing Faculty of Medicine, The University of Hong Kong, Pokfulam, Hong Kong Special Administrative Region, People’s Republic of China; b State Key Laboratory for Emerging Infectious Diseases, Carol Yu Centre for Infection, Department of Microbiology, Li Ka Shing Faculty of Medicine, The University of Hong Kong, Pokfulam, Hong Kong Special Administrative Region, People’s Republic of China; c Department of Clinical Microbiology and Infection Control, The University of Hong Kong-Shenzhen Hospital, Shenzhen, People’s Republic of China; d Department of Medicine and Geriatrics, Princess Margaret Hospital, Hong Kong, Hong Kong Special Administrative Region, People’s Republic of China; e Department of Pathology, Princess Margaret Hospital, Hong Kong, Hong Kong Special Administrative Region, People’s Republic of China; f Department of Medicine, Li Ka Shing Faculty of Medicine, The University of Hong Kong, Pokfulam, Hong Kong, People’s Republic of China

**Keywords:** COVID19, SARS-CoV-2, neutralizing antibody, disease severity, ICU patient

## Abstract

Coronavirus disease 2019 (COVID-19) has a wide spectrum of disease severity from mild upper respiratory symptoms to respiratory failure. The role of neutralizing antibody (NAb) response in disease progression remains elusive. This study determined the seroprevalence of 733 non-COVID-19 individuals from April 2018 to February 2020 in the Hong Kong Special Administrative Region and compared the neutralizing antibody (NAb) responses of eight COVID-19 patients admitted to the intensive care unit (ICU) with those of 42 patients not admitted to the ICU. We found that NAb against SARS-CoV-2 was not detectable in any of the anonymous serum specimens from the 733 non-COVID-19 individuals. The peak serum geometric mean NAb titer was significantly higher among the eight ICU patients than the 42 non-ICU patients (7280 [95% confidence interval (CI) 1468-36099]) vs (671 [95% CI, 368-1223]). Furthermore, NAb titer increased significantly at earlier infection stages among ICU patients than among non-ICU patients. The median number of days to reach the peak Nab titers after symptoms onset was shorter among the ICU patients (17.6) than that of the non-ICU patients (20.1). Multivariate analysis showed that oxygen requirement and fever during admission were the only clinical factors independently associated with higher NAb titers. Our data suggested that SARS-CoV-2 was unlikely to have silently spread before the COVID-19 emergence in Hong Kong. ICU patients had an accelerated and augmented NAb response compared to non-ICU patients, which was associated with disease severity. Further studies are required to understand the relationship between high NAb response and disease severity.

## Introduction

Since emerging in late 2019, the coronavirus disease (COVID-19) has rapidly spread across the world [1]. The World Health Organization declared COVID-19 a pandemic on 11 March 2020. As of 29th June 2020, there are over 10 million laboratory-confirmed cases worldwide with more than 0.5 million deaths. The severe acute respiratory syndrome coronavirus 2 (SARS-CoV-2) predominantly causes respiratory tract infection. It also replicates to higher titers than SARS-CoV in *ex vivo* lung tissue explant cultures [2,3]. Moreover, about 20% of patients experience gastrointestinal symptoms, and SARS-CoV-2 can infect and replicate in human intestinal cell line and organoid [4,5].

Understanding the host immune response to SARS-CoV-2 is critical in deciphering the pathogenesis of COVID-19. We have previously shown that SARS-CoV-2 could stimulate inflammatory mediators in ex vivo lung tissues, though this stimulation is less than that of the 2003 SARS-CoV [2]. In a hamster model, we have demonstrated there is marked cytokine activation and lymphoid atrophy [6]. Recovered hamsters showed a robust production of neutralizing antibody (NAb) [6].

Using enzyme immunoassay, we and others have shown that IgG against SARS-CoV-2 nucleoprotein (NP) and spike protein receptor binding domain (RDB) started to increase during the second week of infection and that most patients had seroconversion by the third week [3,7–9]. NAb response with a titer of at least 1:20 was identified in 91% of patients during the convalescent period [10]. Recent studies of vaccine in non-human primates and monoclonal neutralizing antibodies in ACE2 transgenic mice suggested that neutralizing antibodies are effective for protection against SARS-CoV-2 [11–14]. In this study, we analysed the temporal NAb responses among patients with severe disease and compared this with the responses of patients with mild disease.

## Methods

### Patients

This study consisted of 733 anonymized archived serum samples collected from the biochemistry laboratory and microbiology laboratory as described previously [15]. These specimens were randomly obtained between April 2018 and February 2020 (Supplementary Table S1), and some specimens have been used in our previous study [10].

A total of 50 patients with COVID-19 were included. All patient cases were confirmed by reverse-transcription polymerase chain reaction (RT–PCR) as we described previously [3]. Patients were excluded if serum specimen was not available on or after day 7 of symptom onset. Eleven patients were described in our previous study [3], 32 patients were included in our previous clinical trial [16], and 9 patients were recruited additionally. Clinical and laboratory findings were entered into a predesigned database. Written informed consent was obtained from all patients, except for the 11 patients for whom archived specimens were used [3]. Ethical approval was obtained from the HKU/HA HKW Institutional Review Board (UW 13-265, UW 13-372, UW 18-141) and Kowloon West Cluster Research Ethics Committee (KW/EX-20-038(144-26)).

### Cell culture

HEK-293T, huh7 and Vero-E6 cells were cultured in Dulbecco’s modified Eagle medium (DMEM) with 10% inactivated fetal bovine serum (FBS) (Invitrogen), 100 units/ml penicillin, and 100 μg/ml streptomycin sulfate (Invitrogen). HEK293T-ACE2 cells were cultured in DMEM with 10% FBS, 100 units/ml penicillin, 100 μg/ml streptomycin sulfate, and 1 μg/ml puromycin (Sigma).

### Pseudovirus-based neutralization assay

The neutralizing activity of heat-inactivated patients’ sera was determined using a pseudotype-based neutralization assay as previously described [17]. The pseudotype virus was generated through cotransfection of 293T cells with 2 plasmids, pVax-1-S-COVID19 and pNL4-3Luc_Env_Vpr, carrying the optimized spike (S) gene (QHR63250) and a human immunodeficiency virus type 1 backbone, respectively as we previously described [17,18] (Supplementary Figure S1). Viral supernatant was collected 48 h post-transfection and was frozen at −150°C. The serially diluted serum samples were incubated with 200 TCID50 of pseudovirus at 37°C for 1 h. The serum-virus mixtures were subsequently added into pre-seeded HEK 293T-ACE2 cells. After 48 h, infected cells were lysed to measure luciferase activity using a commercial kit (Promega, Madison, WI). The NAb titer is defined as the serum dilution that resulted in 50% inhibitory concentrations (IC_50_) as determined by log (inhibitor) vs. normalized response – Variable slope model.

### Live SARS-CoV-2-based microneutralization (MN) assay

This MN assay has been previously described by us [3].

### Statistical analysis

Statistical analysis was performed using PRISM 6.0 or SPSS 26.0. Categorical and continuous variables were compared using Fisher’s exact test and Mann–Whitney U test, respectively. Log-transformed NAb titer was used for the comparison of geometric mean titer with the student’s t test and to analyze the correlation between pseudovirus and MN assays by Pearson correlation test. For the purpose of statistical analysis, a value of 25 was assigned for NAb titer <50. NAb titers above the median of all 50 patients were considered to be of high titer, while NAb titers below the median were considered to be of low titer. To determine independent factors associated with NAb titer, backward stepwise regression analysis was used to control confounding factors.

## Results

**Figure 1.**
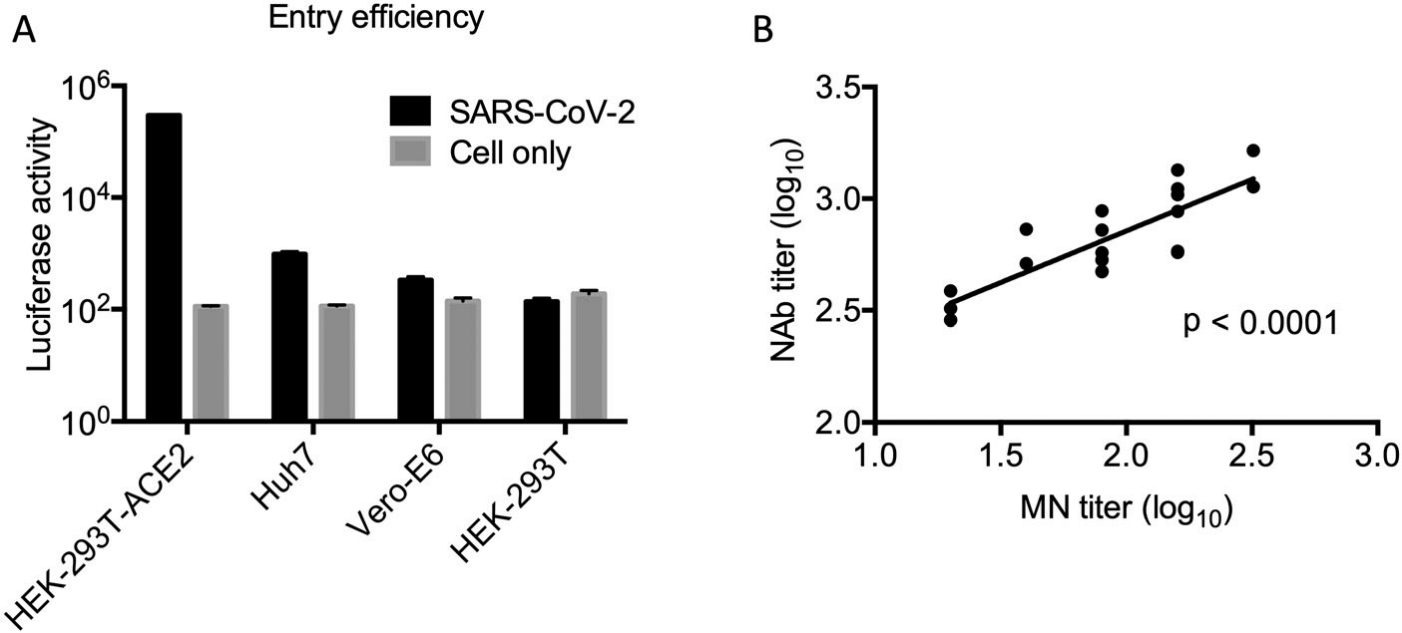
(A) Entry assay of pseudotyped virus. 50 μl pseudovirus was used to infect 2 × 10^4^ HEK293-ACE2, Huh7, Vero-E6 and HEK-293T cells, respectively. Luciferase activity was measured 48 h postinfection using the Promega kit. Triplicates were tested in each experiment. The average values and standard error bars are presented. The experiment was repeated three times with similar results obtained. (B) Comparison of pseudotyped neutralization assay with the live SARS-CoV-2 based MN assay. Log-transformed NAb titers (IC_50_) are presented in the plot. Pearson correlation test results demonstrated a significant positive correlation (*p* < 0.0001) between two assays.

We first developed a single-cycle reporter pseudotyped virus containing a spike glycoprotein of SARS-CoV-2. We examined the entry efficiency of pseudotyped viruses into the Vero-E6, Huh7 and HEK293T-ACE2 cells. We found that pseudotyped viruses were able to infect all target cells, with highest infection efficiency in HEK293T-ACE2 cells (Figure 1). We conducted parallel experiments with HEK-293T cells and found that the pseudotyped viruses did not infect these cells (Figure 1A). Furthermore, we sought to compare pseudotyped neutralization assay with the live SARS-CoV-2 based MN assay, which we have previously described [3]. By testing the same set of 18 patients sera, we found that there was a strong correlation between Log-transformed NAb titers measured by pseudotyped and MN assays by the Pearson correlation test (*p* < 0.0001) (Figure 1B). We discovered that the pseudotyped assay was on average 5.75-fold (range, 2.96- to 9.03-fold) more sensitive than the MN assay.

To assess the starting point of COVID-19 in Hong Kong Special Administrative Region (HKSAR), we applied our assay to 733 anonymized serum specimens. All specimens tested were negative at a dilution of 1:50. Our results, therefore, not only demonstrated the specificity of the pseudovirus NAb assay, but also indicated that SARS-CoV-2 was unlikely to be circulating in HKSAR before its emergence in our patients.

Next, we determined the NAb of 50 COVID-19 patients, including 8 patients admitted to the intensive care unit (ICU) and 42 hospitalized patients who were not admitted to the ICU (Table 1). The median age was 56 years (interquartile range [IQR], 33–62) and 22 (44%) were female. ICU patients were significantly older than non-ICU cases (ICU patients, 63 years [IQR, 57–70] vs non-ICU patients, 49 years [IQR, 30–60], *P* = 0.007). Among the presenting symptoms, dyspnea was significantly more common among ICU patients (ICU patients, 4/8 [50%] vs non-ICU patients, 6/42 [14.3%], *P* = 0.041). For laboratory tests on admission, lymphocyte count (ICU patients, 0.7 × 10^9^/L [IQR, 0.4–0.9] vs non-ICU patients, 1.1 × 10^9^/L [0.8–1.7], *P* = 0.004) and hemoglobin level (ICU patients, 12.9 g/dL [IQR, 12.3–13.3] vs non-ICU patients, 13.9 g/dL [IQR, 12.8–14.9], *P* = 0.003) were significantly lower for ICU patients than those of non-ICU patients. There was also a trend towards a lower platelet count for ICU patients than for non-ICU patients, almost reaching statistical significance (ICU patients, 158 × 10^9^/L [IQR, 137–181] vs non-ICU patients, 190 × 10^9^/L [IQR, 163–261], *P* = 0.061).

**Table 1.** Clinical characteristics of patients in this study.

Characteristics	Admitted to ICU (*n *= 8)	Non-ICU patients (*n *= 42)	*P* value
*Demographic*			
Age, median years (interquartile range)	63 (57–70)	49 (30–60)	0.007
Female	4 (50)	24 (57.1)	0.718
*Chronic comorbidities*			
Hypertension	3 (37.5)	6 (14.3)	0.144
Chronic heart disease	0 (0)	3 (7.1)	1.000
Chronic lung disease	0 (0)	2 (4.8)	1.000
Chronic liver disease	1 (12.5)	0 (0)	0.160
Chronic kidney disease	1 (12.5)	1 (2.4)	0.297
Diabetes mellitus	2 (25)	7 (16.7)	0.623
No chronic comorbidities	3 (37.5)	26 (61.9)	0.255
*Presenting symptoms*			
Fever	7 (87.5)	27 (64.3)	0.409
Dyspnea	4 (50)	6 (14.3)	0.041
Cough	2 (25)	23 (54.8)	0.247
Rhinorrhea	0 (0)	8 (19.0)	0.324
Sore throat	1 (12.5)	9 (21.4)	1.000
Diarrhoea	1 (12.5)	10 (23.8)	0.666
*Blood tests on admission, median, interquartile range*			
Haemoglobin (g/dL)	12.9 (12.3–13.3)	13.8 (12.8–14.9)	0.039
Total white blood cell count (×10^9^/L)	5.8 (3.6–9.0)	5.4 (4.3–6.5)	0.707
Neutrophil count (×10^9^/L)	4.5 (2.3–8.2)	3.7 (2.5–4.4)^a^	0.488
Lymphocyte count (×10^9^/L)	0.7 (0.4–0.9)	1.1 (0.8–1.7)^a^	0.004
Platelet count (×10^9^/L)	158 (137–181)	191 (163–261)	0.061
Urea (mmol/L)	4.5 (3.5–6.0)	4.3 (2.9–5.0)	0.397
Creatinine (μmol/L)	63 (54–92)	79 (66–93)	0.194
Alanine aminotransferase (U/L)	32 (23–41)	27 (19–45)	0.668
*Severity*			
Oxygen supplementation	8 (100)	7 (16.7)	<0.001
Death	1 (12.5)	0 (0)	0.160
Geometric mean neutralizing antibody titer (95% confidence interval)	7280 (1468-36099)	671 (368-1223)	0.002

^a^Neutrophil count and lymphocyte count available for 40 patients.

**Figure 2.**
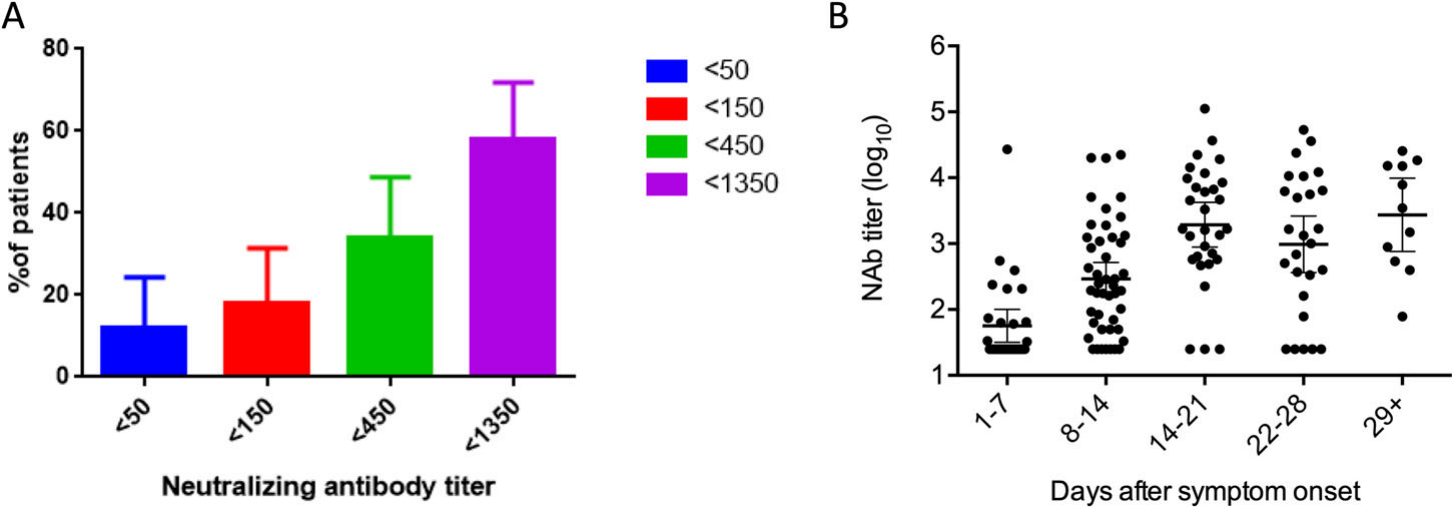
Neutralizing antibody profiles of patients determined by pseudotyped neutralization assay. (A) Proportion of patients with low neutralizing antibody titer. Error bar indicates 95% confidence interval. (B) Peak levels of neutralizing antibodies in patients at different time points after symptoms onset. Dots represent the NAb titer in patient serum. Geometric mean of the NAb titer is shown by a line. Error bar indicates 95%.

Overall, the peak geometric mean of NAb titer was 982 (95% confidence interval [CI], 541-1784). Out of these 50 patients, 6 (12%) had a NAb titer of <50 and 17 (34%) had NAb titers of <450 (Figure 2A). The NAb titer increased from 1st to 3rd week (Figure 2B).

**Figure 3.**
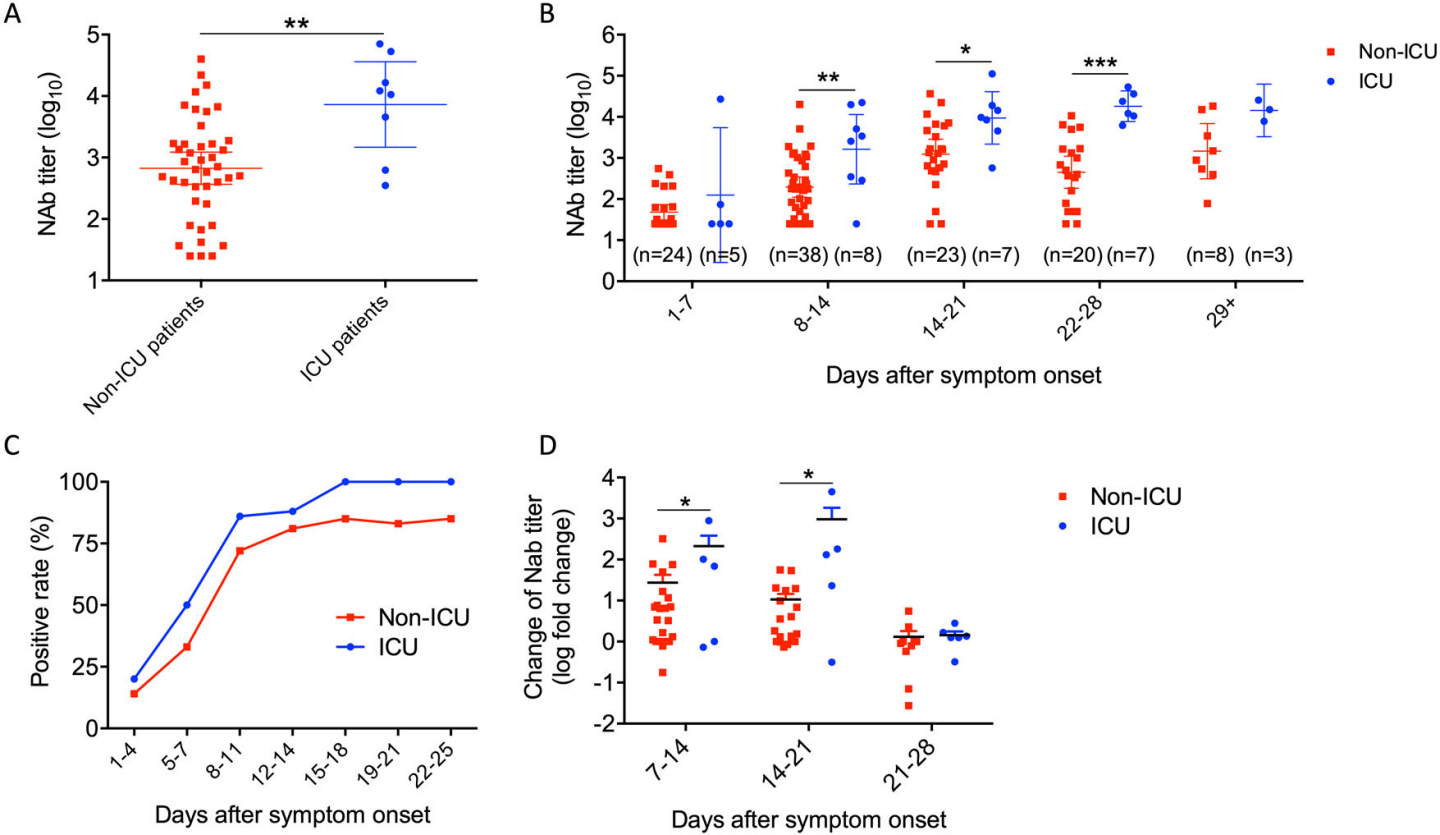
Differences of neutralizing antibody titers between ICU and non-ICU patients. (A) Comparison of peak geometric mean titers between ICU and non-ICU patients. (B) Comparison of geometric mean titers between ICU and non-ICU patients at weekly intervals after symptoms onset. The highest titer during each weekly period was presented. (C) Comparison of seropositive rates between ICU and non-ICU patients. A serum specimen is considered to be seropositive if the defined 50% inhibitory concentration (IC_50_) value was above 1:50. (D) Comparison of Nab titer change between ICU and non-ICU patients at weekly intervals after symptoms onset. Fold change was calculated using the highest titer from each time period against the highest titer from prior week. The error bar indicates 95% confidence interval. Unpaired student’s *t*-test was used. *P <* *0.05, **P <* *0.01, ***P <* *0.001.

Next, we compared ICU and non-ICU patients. ICU patients had significantly higher peak NAb titer than non-ICU patients (ICU patients, 7280 (95% confidence interval [CI] 1468-36096); non-ICU patients, 671 [95% CI, 368-1223]) (Figure 3A). ICU patients also had significantly higher NAb titer than non-ICU cases as early as 8–14 days after symptoms onset (Figure 3B). ICU patients had higher positive rates of serum diagnosis than non-ICU patients from 1st week onwards (Figure 3C). Furthermore, NAb increased significantly earlier among ICU patients than non-ICU patients (Figure 3D). The median number of days after symptoms onset to reach the peak Nab titer was also shorter among ICU patients (17.6) than non-ICU patients (20.1).

To determine the risk factors for high NAb titer, we performed both univariate and multivariate analysis. In the univariate analysis, significantly more patients in the high titer group required oxygen supplement than those in the low titer group (52% [13/25] vs 8% [2/25], *P* = 0.001) (Table 2). Fever was significantly more frequent for high titer group than low titer group (84% [21/25] vs 52% [13/25]). Lymphocyte (0.9 [IQR, 0.7–1.5] vs 1.2 [IQR, 0.9–1.8]; *P* = 0.033) and platelet counts (164 [IQR 138–243] vs 199 [172–260], *P* = 0.021) were significantly lower for high titer than low titer group. In the multivariate analysis, only fever (*P* = 0.045) and oxygen supplementation (*P* = 0.010) were independent factors associated with NAb titer.

**Table 2.** Univariate analysis to determine factors associated with peak neutralizing antibody titer.

	High titer group (*n *= 25)	Low titer group (*n* = 25)	*P* value
*Demographic*			
Age, median years (interquartile range)	57 (47–64)	51 (29–61)	0.065
Female	10 (40)	12 (48)	0.776
*Chronic comorbidities*			
Hypertension	5 (20)	4 (16)	1.000
Chronic heart disease	3 (12)	0 (0)	0.235
Chronic lung disease	0 (0)	2 (8)	0.490
Chronic liver disease	1 (4)	0 (0)	1.000
Chronic kidney disease	0 (0)	2 (8)	0.490
Diabetes mellitus	5 (20)	4 (16)	1.000
No chronic comorbidities	13 (52)	16 (64)	0.567
*Presenting symptoms*			
Fever	21 (84)	13 (52)	0.032
Dyspnea	5 (20)	5 (20)	1.000
Cough	9 (36)	16 (64)	0.089
Rhinorrhea	3 (12)	5 (20)	0.702
Sore throat	5 (20)	5 (20)	1.000
Diarrhea	5 (20)	6 (24)	1.000
*Blood tests on admission, median, interquartile range*			
Hemoglobin (g/dL)	13.3 (12.6–14.5)	14.0 (12.5–15.0)	0.232
Total white blood cell count (×10^9^/L)	5.1 (3.5–6.1)	5.7 (4.8–6.9)	0.135
Neutrophil count (×10^9^/L)	3.9 (2.3–4.5)	3.5 (2.5–4.8)	0.613
Lymphocyte count (×10^9^/L)	0.9 (0.7–1.5)	1.2 (0.9–1.8)	0.033
Platelet count (×10^9^/L)	164 (138–243)	199 (172–260)	0.021
Urea (mmol/L)	4.1 (3.5–5)	4.3 (2.9–5.0)	0.719
Creatinine (μmol/L)	76 (64–99)	78 (64–90)	0.861
Alanine aminotransferase (U/L)	33 (22–46)	23 (17–44)	0.156
*Severity*			
Oxygen supplementation	13 (52)	2 (8)	0.001
ICU admission	6 (24)	2 (8)	0.247
Death	0 (0)	1 (4)	1.000

## Discussion

Knowledge regarding the NAb response for COVID-19 patients is critical for understanding the host humoral immune response towards SARS-CoV-2 and the pathogenesis of COVID-19. In this study, the absence of NAb in the serum of over 733 HKSAR residents indicates that SARS-CoV-2 is unlikely to have spread silently in Hong Kong before its emergence in COVID-19 patients. Furthermore, by comparing ICU and non-ICU patients, we have shown that NAb response rose significantly earlier and to a much greater extent in severe patients than in mild patients. Multivariate analysis showed that oxygen requirement and fever were the only factors associated with a higher NAb response. The oxygen requirement signifies the extent of local lung damage due to the infection by SARS-CoV-2, while the fever response indicates the systemic inflammatory reaction by the immune system of the host towards the virus.

We have demonstrated that patients with severe disease developed a faster and higher level of NAb response. Previously, we and others showed that 2003 SARS-CoV patients who died also had a more rapid NAb response [19,20]. There are several reasons why the faster NAb response did not ameliorate the severe disease. First, there can be overwhelming virus-induced damage in the lungs, which exacerbates proinflammatory cytokine response [21,22]. Since the antibody only neutralizes the virus, the inflammation triggered by virus-induced damage cannot be dampened by a NAb response. In our hamster model, we have demonstrated that there is extensive diffuse alveolar damage and apoptosis in the lung, which was associated with significant cytokine activation [2]. Second, we reported that patients with COVID-19 had the highest viral load near symptoms presentation and rapid antibody development could enhance macrophage-mediated acute lung injury [3,17]. High NAb titer in ICU patients might be due to higher viral/antigen loads during acute SARS-CoV-2 infection. Third, we have previously shown that the anti-spike protein antibody, which contains potent receptor binding domain-specific NAb [23], can worsen disease in a macaque model by skewing inflammation-resolving responses [17].

Several studies have evaluated the kinetics of antibodies against the SARS-CoV-2 NP or spike protein. Previous studies have shown that the antibody titer against these proteins were higher among patients with severe disease than those with mild disease [7]. However, these antibodies that bind to NP or spike protein may not be neutralizing. In a study by Okba et al., it was shown that one patient with severe disease had a faster and more augmented NAb response than two patients with mild disease [24]. Wang et al. has also shown that patients with severe disease had high titers of NAb, but the number of patients were not shown [25]. During our manuscript revision, a preprint paper indicated that SARS-CoV-2 neutralizing antibody responses are more robust in patients with severe disease [26]. In a convalescent plasma transfusion study, 9 out of 10 severe patients (one unavailable) had actually self-developed NAb responses before the treatment [27]. Since 4 severe cases had the same high NAb titer (1:640) even before and also after the transfusion, the therapeutic benefits of NAb remains to be investigated.

In our cohort, 12% of patients had a neutralizing titer below 50 and 34% had a NAb titer below 450. The inability to mount a high antibody titer corroborates with the results from a study by Wu et al., which also showed that 5% of patients had undetectable pseudovirus NAb levels [28]. Though the cell mediated immune response or cytotoxic lymphocyte response were not measured in these patients, the low level of NAb suggests that some patients may be susceptible to re-infection in the future. These patients may also have a chance for longer period of viral shedding. Currently, the protective NAb titer has not been established. Therefore, it would be important to follow-up patients to assess the protective NAb titer level, which may have significant implication for vaccine development.

We have used a pseudovirus neutralization assay. The main advantage of using pseudovirus assay is that the experiment can be performed in laboratories of biosafety level 2 instead of biosafety level 3. Furthermore, the pseudovirus neutralization assay is a high throughput assay and therefore a large number of serum specimens can be assessed simultaneously. Results from pseudovirus neutralization assays are also highly reproducible [29].

Measuring NAb is especially required for the screening of patients as convalescent plasma donors. It is also important to screen the collected convalescent plasma of patients who require passive immunization. The use of convalescent plasma has been reported for 10 severe patients, which showed beneficial effect [27,30]. Specially, there was better oxygenation, decreased inflammatory markers, and radiological improvement after patients were treated with infusion of convalescent plasma. Further studies should be performed to understand the optimal timing of convalescent plasma administration, which may improve the outcome of severe COVID-19 patients and minimize the risk of immunopathology.

However, antibodies may also be as dangerous as they are helpful. Although NAb response is important in vaccine-induced immune response, as demonstrated in influenza vaccine trials, antibodies can also worsen disease cases, especially for dengue virus infection. Therefore, the next step is to determine why patients still have worsened disease despite the rapid development of high titer of NAb responses. Understanding NAb response is important clinically, especially for the use of convalescent plasma or hyperimmune globulin therapy. Further studies on whether treatment with neutralizing antibodies is useful in earlier stages of disease remains to be carefully conducted. Specifically, it is important to know whether such treatment should be started earlier, when the inflammatory damage to the lungs is still limited, or should be started later, when the amount of virus is already overwhelming, leading to further lung damage mediated by complement fixation due to excessive antibody–antigen complex formation. For example, a recent study has demonstrated that convalescent plasma or hyperimmune intravenous immunoglobulin against 2009 pandemic influenza H1N1 is only useful within 5 days of symptom onset [31]. In addition, convalescent plasma or monoclonal antibodies especially derived from severe patients should be carefully studied for therapeutic use.

There are several limitations to this study. First, all patients included in this study were adults. The NAb response in children should be compared. Second, this study assessed the NAb response of patients during the acute and subacute phase of infection. Their long term antibody response is still not known.

Our study has demonstrated the association between clinical severity and NAb response. Further studies are required to dissect the immunological events that lead to heightened NAb response. In particular, whether or not neutralizing antibodies themselves can mediate disease severity remains to be investigated.

## Supplementary Material

Supplementary_Table_S1_0509_0906.doc
