# Supplementary material for: High neutralizing antibody titer in intensive care unit patients with COVID-19
Source: Emerg Microbes Infect. 2020 Jul 20;9(1):1664–70. doi: 10.1080/22221751.2020.1791738 (PMC7473321; doi:10.1080/22221751.2020.1791738)
Supplement: Supplementary_Table_S1_0509_0906.doc [file TEMI_A_1791738_SM8055.doc]

**Supplementary Table S1.** Number of anonymous archived serum specimens in each age group.

| **Age group (years)** | **2018**  **Apr-Jul** | **2019**  **Jan-Jun** | **2019**  **Jul-Dec** | **2020**  **Jan** | **2020**  **Feb** |
| --- | --- | --- | --- | --- | --- |
| 0-9 | 8 | 8 | 2 | 8 | 0 |
| 10-19 | 5 | 23 | 18 | 18 | 1 |
| 20-29 | 5 | 21 | 17 | 26 | 10 |
| 30-39 | 2 | 18 | 21 | 23 | 24 |
| 40-49 | 2 | 22 | 24 | 22 | 26 |
| 50-59 | 4 | 20 | 22 | 25 | 25 |
| 60-69 | 0 | 18 | 23 | 27 | 20 |
| 70-79 | 10 | 19 | 23 | 20 | 24 |
| 80 or above | 7 | 13 | 29 | 29 | 21 |
| Total | 43 | 162 | 179 | 198 | 151 |
